# The Functional Benefits of Preserving the Proximal Lower Leg Even in Total Necrosis of the Lower-Leg Muscles

**Published:** 2020-08-31

**Authors:** Itaru Tsuge, Michiharu Sakamoto, Jun Arata, Naoki Morimoto

**Affiliations:** ^a^Department of Plastic and Reconstructive Surgery, Graduate School of Medicine, Kyoto University, Kyoto, Japan; ^b^Department of Plastic and Reconstructive Surgery, National Hospital Organization Kyoto Medical Center, Kyoto, Japan

**Keywords:** amputation, gastrocnemius, lower leg, prosthetic leg, soleus

## DESCRIPTION

**Figure 1:**
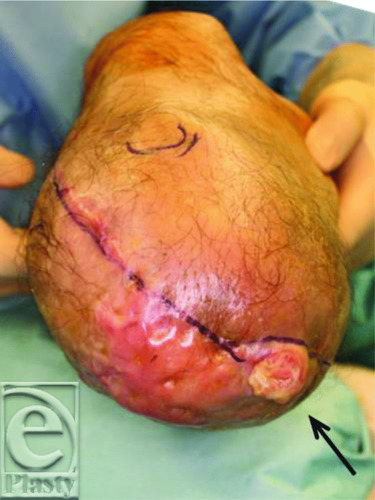
Preoperative findings. The black arrow indicates an ulcer on the right lower leg.

**Figure 2:**
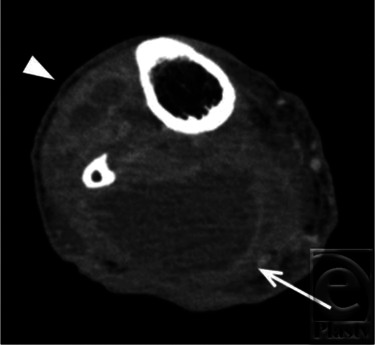
Contrast computed tomographic scan shows fluid collection in both the posterior segment (white arrow) and the anterior segment (white arrowhead).

**Figure 3:**
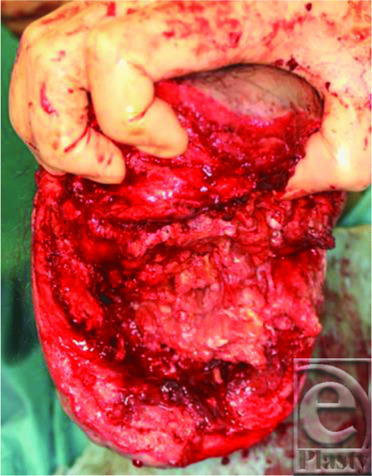
Intraoperative findings. The soleus and gastrocnemius muscles are necrotized with infection.

A 43-year-old man was introduced to us for the treatment of an infected wound after below-knee (B-K) amputation (Fig 1). Four months previously, he had experienced sudden cardiogenic shock and was transferred to the emergency department of another hospital. Under a diagnosis of fulminant myocarditis, percutaneous cardiopulmonary support (PCPS) from the right femoral artery was applied. His general condition gradually improved; however, PCPS caused right lower-limb ischemia and resulted in B-K amputation. After surgery, severe infection occurred and above-knee (A-K) amputation was recommended for an early cure. At the first visit to our hospital, contrast computed tomographic scan showed fluid accumulation at both the posterior and anterior tibias (Fig 2). We opened the scar and found that the ulcer had reached the popliteal fossa, with a large amount of yellow discharge. All of the lower-leg muscles, including the gastrocnemius and soleus muscles, were necrotic and were removed (Fig 3).

## QUESTIONS

1. How can this stump be treated?
2. What is the preferred level of amputation?
3. What are the risks of preserving the proximal lower-leg region?
4. What measures are available for preventing the development of pressure injuries?

## DISCUSSION

**Figure 4:**
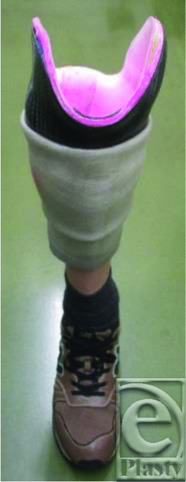
All-surface loading prosthesis.

**Figure 5:**
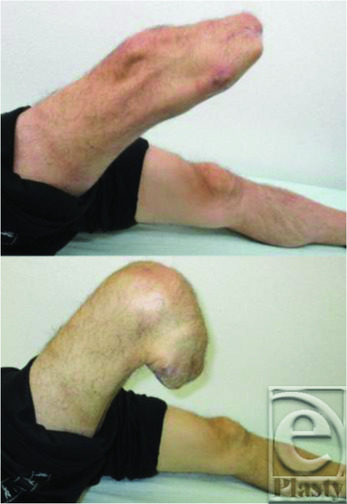
Postoperative findings. The range of motion is from 0° to 130°.

The opened wound was washed and treated with silver sulfadiazine cream. On the 17th postoperative day, we initiated negative pressure wound therapy (NPWT) at −125 mm Hg, under a diagnosis of disappearance of infection. After 8 days of NPWT, the wound showed good granulation. Successive treatment using basic fibroblast growth factor (bFGF) and bucladesine sodium ointment was performed to promote capillary formation and accelerate wound healing. We observed epithelialization of the ulcer on 35th postoperative day. Considering the bone stump was covered only by scar, we initially used an Iceross Dermo cushion liner (Ozur, Iceland); however, the stimulation caused by the loss of soft tissue made continuous wearing difficult. Thus, the liner was changed to a 6Y92 Copolymer Liner (Otto Bock, Germany), which he was able to wear continuously. Next, a patella tendon-supporting load-relieving brace was made; however, the stump moved in the socket, resulting in skin damage. In contrast, an all-surface loading prosthesis fit well (Fig 4). At 18 months after the surgery, the range of motion of the right knee joint was 0° to 130° (Fig 5). The muscle strength reached MMT-5. He was able to go to work on foot and climb stairs.

Surgeons need to choose whether to perform A-K amputation, to achieve early wound resolution, or to preserve the B-K limb, which results in a better function. In B-K amputation, covering a tibial stump with well-vascularized soleus and gastrocnemius muscles is important for preventing the development of pressure ulcers and preserving the knee joint function.^1^ Thus, we often consider A-K amputation when extensive necrosis of the lower-leg muscles occurs. However, recent advances in surgical wound care, such as NPWT and bFGF, have enabled the curative treatment of severely infected and damaged wounds. We showed that preserving the proximal lower leg had a functional benefit, even with the complete loss of the lower-leg muscles, including the soleus and gastrocnemius muscles.

**Figure 6:**
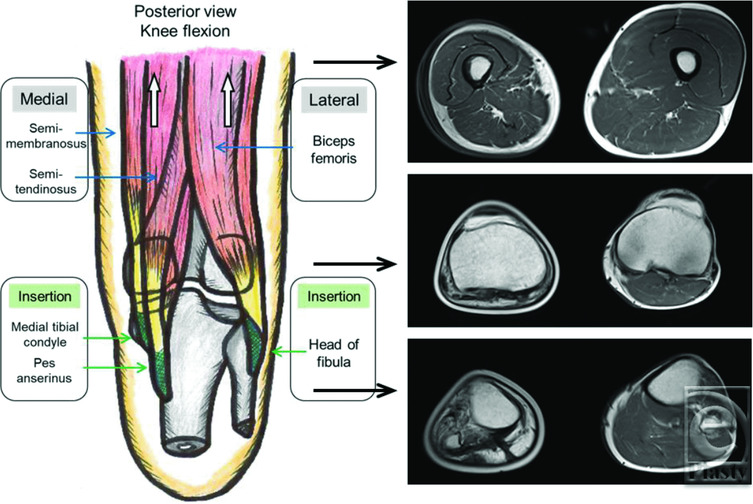
A schematic illustration of the right lower leg and magnetic resonance imaging findings.

We were anxious about 2 risks when preserving the proximal lower-leg region. The first risk was that the preserved knee would lose its function and cause increased instability during walking. A-K amputation is associated with high energy consumption and difficult rehabilitation, even in younger patients.^2^ However, the decreased knee function caused by either joint contracture or a loss of muscle force makes prosthetic training more difficult. Thus, rehabilitation of the hamstrings (biceps femoris, semitendinosus, and semimembranosus muscles), which are the sources of force in knee flexion, is important.^3^ Preserving the head of the fibula, the medial tibial condyle, and the pes anserinus is also essential for knee flexion. We started training using the posterior thigh muscles in the early postoperative period to preserve the knee joint function. NPWT for open wounds worked well for rehabilitation without a rest period. Postoperative magnetic resonance imaging at 18 months revealed that all lower-leg muscles were lost until the right popliteal fossa (Fig 6); however, the knee function was well maintained. The left quadriceps femoris showed compensatory development.

The second risk was the potential for the development of pressure ulcers on the tibial stump because of lost muscle coverage. The management of stumps has been improved with recent silicon liners. The sleeve-like structure lined with silicone inside can reduce friction between skin and socket while maintaining an excellent suspension action.^4^ In this case, deep pressure ulcers were prevented by maintaining the skin perception around the stump. We first considered covering the tibia with sufficient soft tissue such as a free latissimus dorsi flap or a reverse anterolateral thigh flap.^5^^,^^6^ However, these flaps would have resulted in the loss of skin perception of stump and the development of deep ulcers.

## SUMMARY

Even if all of the lower-leg muscles are lost, we can preserve a functional lower leg. It is necessary to understand the advantages and disadvantages of active preservation of lower-limb length.
